# Sequential structural changes in the monomeric LOV histidine kinase EL346 revealed by transient infrared spectroscopy

**DOI:** 10.1039/d6cp03453e

**Published:** 2026-10-22

**Authors:** Philipp Janke, Raoul E. Herzog, Philipp J. Heckmeier, Peter Hamm

**Affiliations:** a Department of Chemistry, University of Zurich Winterthurerstrasse 190 CH-8057 Zurich Switzerland philipp.janke@chem.uzh.ch peter.hamm@chem.uzh.ch

## Abstract

Light-oxygen-voltage (LOV) domains are flavin-binding photoreceptors that mediate cellular responses to blue light. They often act as a structural switch in multidomain proteins to facilitate signaling, where the precise mechanism is dictated by the specific multidomain architecture. A few different LOV signaling models have been categorized, but an understanding of LOV signaling remains a challenge in the field. We studied the multidomain photoreceptor EL346, which couples a LOV domain to a histidine kinase effector domain. Its unique monomeric structure sets it apart from classical, dimeric LOV histidine kinases that act through a structurally entirely different pathway. We present transient infrared spectroscopy data that reveal the timescales on which activation takes place. By studying the isolated core LOV domain, we were able to isolate signal contributions originating from the histidine kinase output domain. The data reveal α-helical structural rearrangements that appear concomitantly with adduct formation at ∼10 µs. We identified a second kinetic step at ∼3 ms, where α-helical elements undergo a final structural adaptation. Our data put the sequence of structural events during the activation of EL346 into context with other LOV multidomain signaling modes, supporting a mechanism similar to *As*LOV2, where signaling proceeds *via* a helix that is bound on the β-sheet surface of the LOV domain. The data add to the known catalog of different LOV signaling modes and contribute to a better understanding of signal transmission between the receptor and effector domains.

## Abstract 2

Transient infrared spectroscopy of the LOV histidine kinase EL346 reveals microsecond-to-millisecond structure changes upon blue-light activation. Signal transduction proceeds through a central α-helical linker in a sequence of kinetic steps.

## Graphical abstract

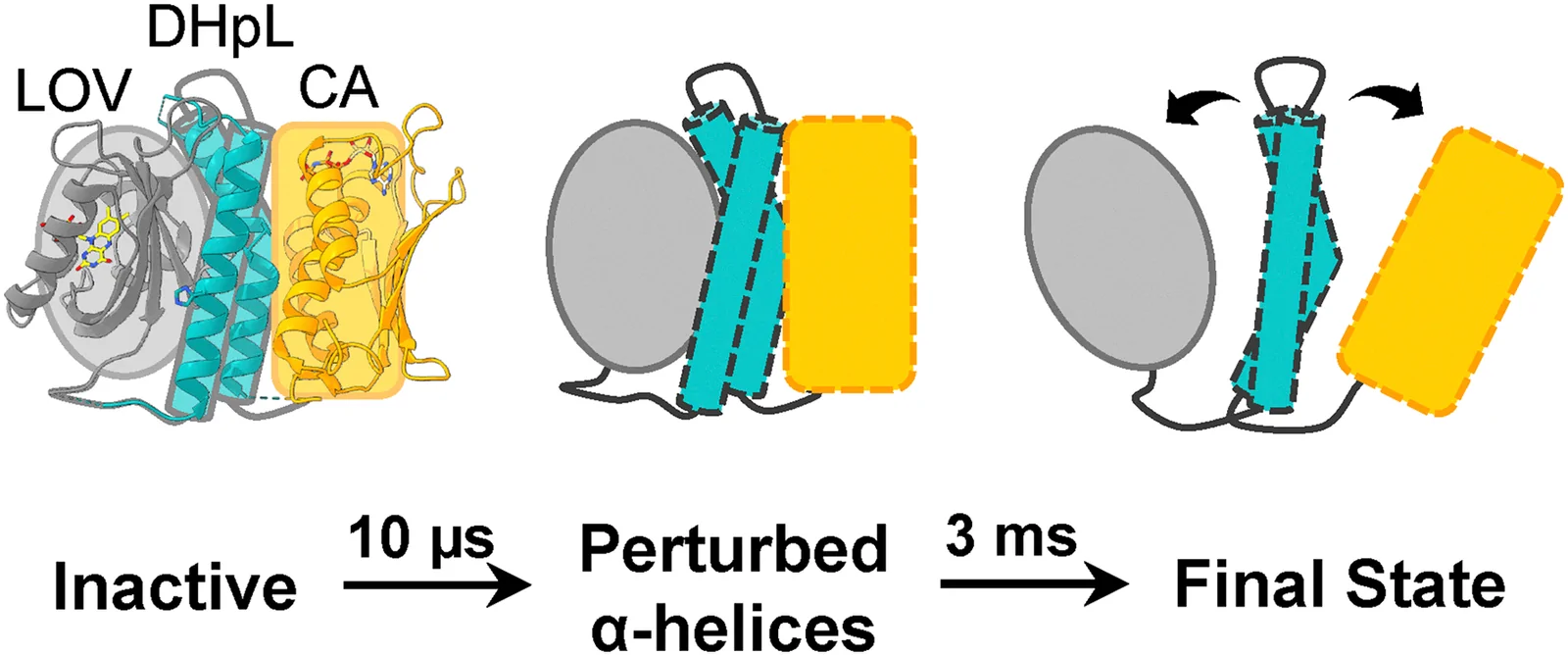

## 1. Introduction

Light-oxygen-voltage (LOV) domains are photoreceptor proteins that use a flavin cofactor, most commonly flavin mononucleotide (FMN), to mediate cellular responses to blue light.^1^ While the LOV domain and its photochemistry are highly conserved, LOV domains are often employed as part of multidomain proteins in prokaryotes, fungi and plants,^2,3^ where they can facilitate signal transduction through a wide range of different mechanisms, which can be roughly categorized into different signaling models.^1^ The *As*LOV2 protein of phototropin is one of the most widely studied systems, where the LOV domain binds directly to a terminal helix, releasing it after light activation (Fig. 1a).^4^*As*LOV2 has been extensively studied by NMR,^4^ transient infrared (IR) spectroscopy,^5,6^ and time-resolved X-ray liquidography,^7^ revealing detailed insight into the signal transduction mechanism. In contrast, dimeric LOV proteins can achieve signaling through helical rotations of effector domains that extend away from the LOV domains, as exemplified by YtvA and YF1 (Fig. 1b).^8–11^ Especially the artificially constructed protein YF1 represents an interesting model system for dimeric LOV signaling.^10^ It consists of a LOV domain fused to a histidine kinase (HK) domain,^12,13^ successfully combining them into a functional, light-switchable enzyme. Through mutagenesis,^10,11^ crystallography,^11^ small-angle X-ray scattering,^14^ electron paramagnetic resonance (EPR) spectroscopy,^15^ and transient IR spectroscopy,^16^ the signaling mechanism was studied in great detail. In YF1, structural changes in the photo-activated LOV domains propagate to the effector domains through supercoiling of parallel coiled-coil linkers, the Jα-helices. As a result, the steric accessibility of residues relevant for the histidine kinase function is altered, modulating the overall enzymatic activity.^13–15,17^ Yet other LOV proteins act through conformational changes that modulate or free binding interfaces, causing dimerization or unbinding and thereby enabling control of their signaling output. Examples include VVD,^18^ EL222,^19^ aureochromes^20^ and *Rs*LOV.^21^ With only a limited number of well-understood examples, the study of LOV multidomain signaling remains a challenge in the field.

**Fig. 1:**
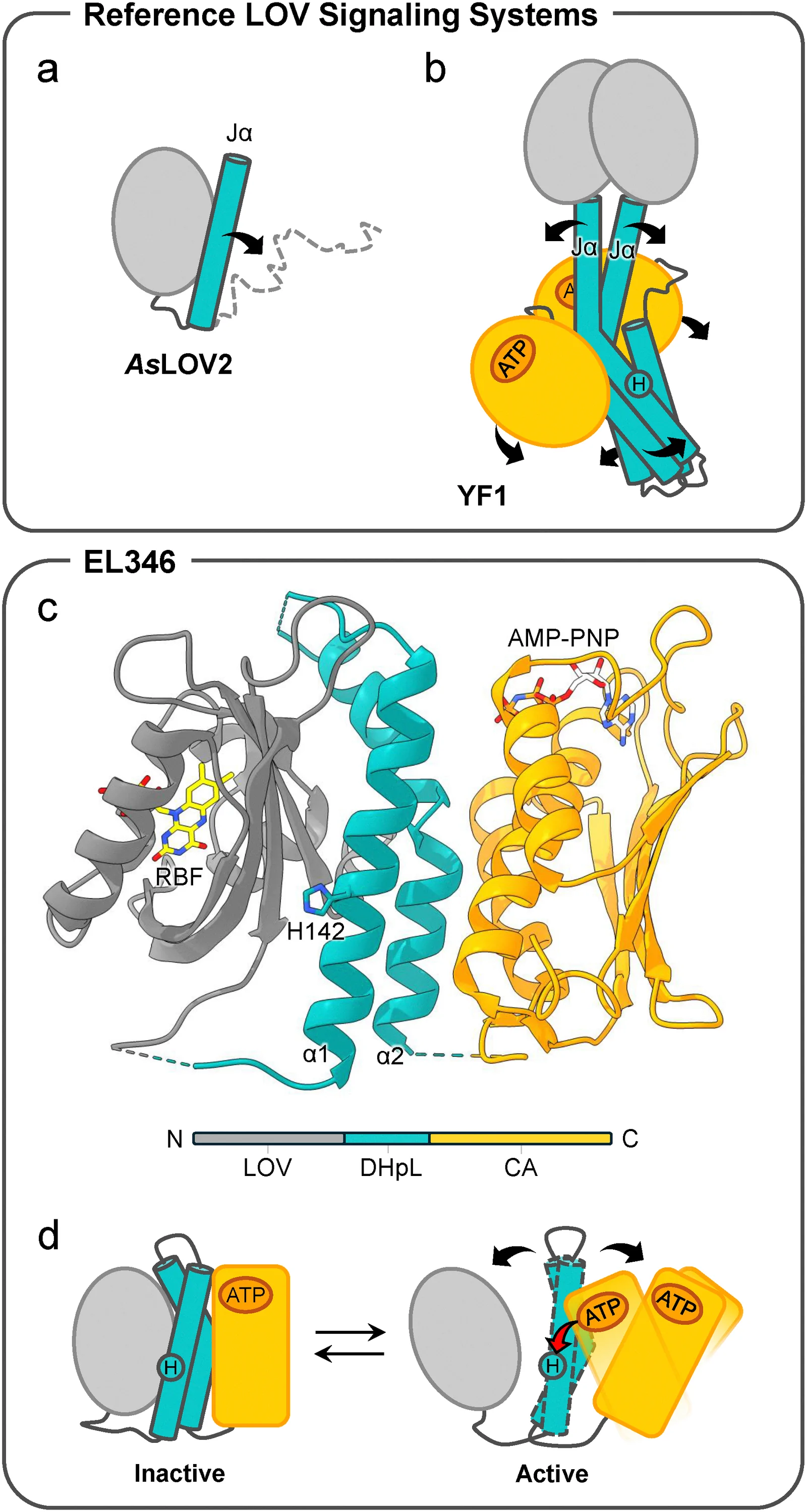
(a) Crystal structure of EL346 (PDB: 4R3A), featuring the LOV (gray), DHpL (cyan) and CA (orange) domains. The riboflavin (RBF) cofactor, the active site histidine (H142) and the bound ATP-analogue AMP-PNP are shown. Dashed lines bridge unresolved residues in the crystal structure. (b) Previously proposed activation mechanism of EL346. The design was adapted from ref. 22. (c) Dark structure of *As*LOV2, with the Jα-helix (blue) bound on the β-sheet surface of the LOV domain (gray). (d) Dark structure of YF1, which adopts a dimeric structure, with the Jα-helices extending into a four-helix bundle (blue) and the CA domain (orange).

In this paper, we present data on the LOV multidomain protein EL346, which adopts a unique structure that is distinct from other LOV multidomain architectures, raising questions about its particular mode of LOV signaling. Extensive work has previously been carried out by the Gardner group, revealing detailed insight into the structure and activation mechanism.^22–25^ EL346 is a monomeric LOV-histidine kinase, and features a LOV domain that is directly bound to its two N-terminal α1 and α2-helices through a large binding interface. These helices, also termed the dimerization/histidine phosphotransfer-like (DHpL) domain, are flanked by both the LOV domain and the catalytic/ATP-binding (CA) domain on either side, holding the ensemble together (Fig. 1c).^22^ As a result of this bound conformation, the protein is mostly inhibited, and the CA domain is not free to phosphorylate the active-site histidine in the DHpL domain.

A striking similarity between EL346 and *As*LOV2 is already discernible from the dark-state structures, suggesting that both of them mediate signaling through α-helical structures bound to the β-sheet interfaces of the respective LOV domains. Further, EL346 can be understood as a variation on the theme of YF1. Contrasting the dimeric LOV-histidine kinase architecture of YF1, where the LOV domains bind to each other and the central α-helical domain dimerizes to form a coiled-coil, the LOV domain in EL346 directly binds its α-helical domain. By its unique architecture, it represents an interesting realization of a LOV-histidine kinase that diverges both in structure and mechanism from dimeric LOV-histidine kinases, such as YF1.

The Gardner group showed that upon light-activation, EL346 exhibited increased mobility and disorder in ^15^N/^1^H transverse relaxation-optimized spectroscopy (TROSY) and limited proteolysis,^22^ together with increased solvent accessibility of the DHpL domain, revealed by hydrogen–deuterium exchange by mass spectrometry (HDX-MS).^24^ A structural model emerged, where the binding interfaces are remodeled and the attractive interactions weaken, releasing the CA domain and facilitating activation. With its gained mobility, the CA domain is able to carry out phosphotransfer from a bound ATP molecule to the active site histidine, located in the DHpL domain (Fig. 1c). Further, site-directed spin-labeling (SDSL) and double electron–electron resonance (DEER) EPR spectroscopy revealed that EL346 exists in multiple active and inactive conformations in both the dark and light. The equilibrium between these conformations is modulated by activation, leading to a 1.6-fold activity enhancement. Currently, the sequence of events during the activation process remains an open question, which is only accessible by experimental methods with high time resolution.

In this paper we present transient IR spectroscopy data of EL346. By revealing the timescales of structural rearrangements, we place EL346 in the context of other LOV multidomain signaling modes. We draw a direct comparison between YF1, *As*LOV2 and EL346, yielding spectroscopic insights into the different LOV signaling modes, with YF1 acting through rotation of its coiled-coil linker,^10,11,16^ and EL346 somewhat resembling the *As*LOV2-like signaling mode, where the direct binding of a helical domain to the LOV domain is modulated.

## 2. Methods

### 2.1. Protein production and isolation

Protein expression was carried out in CpxRibF cells (CmpX13 ribC::cat-ribF), provided by Tilo Mathes *et al.*^26^ The sequences for EL346 and LOV_(EL346)_ (amino acids 1–134 of the full-length construct) were encoded on pET-30a(+) plasmids (synthesized by GenScript, NJ, USA), and cells were transformed using electroporation. Cells were grown in lysogeny broth (LB) medium at 37 °C until they reached an OD_600_ of 0.6. Protein expression was induced with 1 mM isopropyl-β-d-1-thiogalactopyranoside (IPTG), riboflavin was added to the medium, and cells were grown overnight at room temperature. After harvesting by centrifugation, cells were sonicated and target protein was isolated *via* Ni-affinity chromatography and subsequently desalted into sample buffer (50 mM Tris–HCl, 100 mM NaCl, pH 8) using size-exclusion chromatography. Sample purity and integrity were validated by mass spectrometry. Samples were concentrated to ∼1 mM for infrared spectroscopy, and buffer exchange to D_2_O was carried out by lyophilization. For sample preparation with the ATP-analogue, adenylyl-imidodiphosphate (AMP-PNP) (Tocris Bioscience) and MgCl_2_ (ITW Reagents) were added directly to the sample, to a final concentration of 5 mM each.

### 2.2. Transient infrared spectroscopy

Transient infrared data from picoseconds to ∼1 s were recorded on a setup consisting of two electronically synchronized lasers, as recently described in our previous work.^27^ Briefly, infrared pulses centered at ∼1650 cm^−1^ were generated by a 100 kHz Pharos system, together with an Orpheus-MIR OPA and a Lyra DFG stage (Light Conversion). Visible pump pulses at 420 nm were obtained by frequency-doubling a 1 kHz Ti:Sa laser system (Mai Tai and Spitfire, Spectra Physics). The visible-pump pulse (*ca.* 200 µm FWHM) and the IR-probe (80 µm FWHM) were overlapped in the sample, with the IR-reference (80 µm FWHM) offset by *ca.* 500 µm. Detection was realized using a spectrograph and a 2 × 32 pixel MCT detector, achieving a spectral resolution of *ca.* 6 cm^−1^ per pixel, and employing custom measurement electronics.^28^ A sample cell connected to a custom-built stopped-flow circuit was used to exchange fresh sample before every excitation shot, as described in our previous work.^16^ To achieve good signal-to-noise for both early and late timescales, two separate measurements with varying repetition rates were recorded, with maximum delays of 50 ms and 1 s, respectively. The two datasets were then combined by blending them into each other using a linear gradient from 30 µs to 25 ms, as described before,^27^ with the combined dataset used for analysis extending to ∼600 ms.

### 2.3. Fourier-transform infrared spectroscopy

FTIR spectra were collected on a Vertex 80v FTIR spectrometer (Bruker Corp. Billerica, MA, USA) with an MCT detector (Kolmar Technologies, Newburyport, MA, USA). Samples were provided using the same stopped flow circuit as for transient IR experiments, and the FTIR instrument aperture was set to 3 mm. After collection of dark spectra, the sample was excited with a GaN multimode laser diode (PLPT9 450D_E, Osram Opto Semiconductors), providing a ∼60 mJ pulse on a ∼5 mm spot for activation. Subsequently, the light spectrum was collected.

## 3. Results

In this study, we recorded transient IR data of the full-length LOV-histidine kinase EL346 in response to activation with blue light (Fig. 2a). The data cover the amide I and amide II regions between 1550 cm^−1^ and 1740 cm^−1^, spanning picoseconds to 600 ms. Typical LOV domain photophysics are characterized by absorption of the flavin cofactor to an excited singlet state, which is followed by intersystem crossing (ISC) to a triplet state. From there, a covalent bond between a conserved cysteine SH group and the C4a atom of the flavin cofactor is formed. This photoadduct formation involves the transfer of a proton to the flavin N5 atom, and the resulting geometry and altered hydrogen bonding network facilitate conformational changes in the LOV domain.^29^ The transient IR data were globally fitted to a sequential model, producing the time constants *τ*_1_ ≈ 2 ns for the decay of the initially excited singlet state into the triplet state of the riboflavin cofactor (RBF), and *τ*_2_ ≈ 9 µs for the formation of the cysteine adduct, matching typical flavin photophysics of LOV domains.^1,3,29,30^

**Fig. 2:**
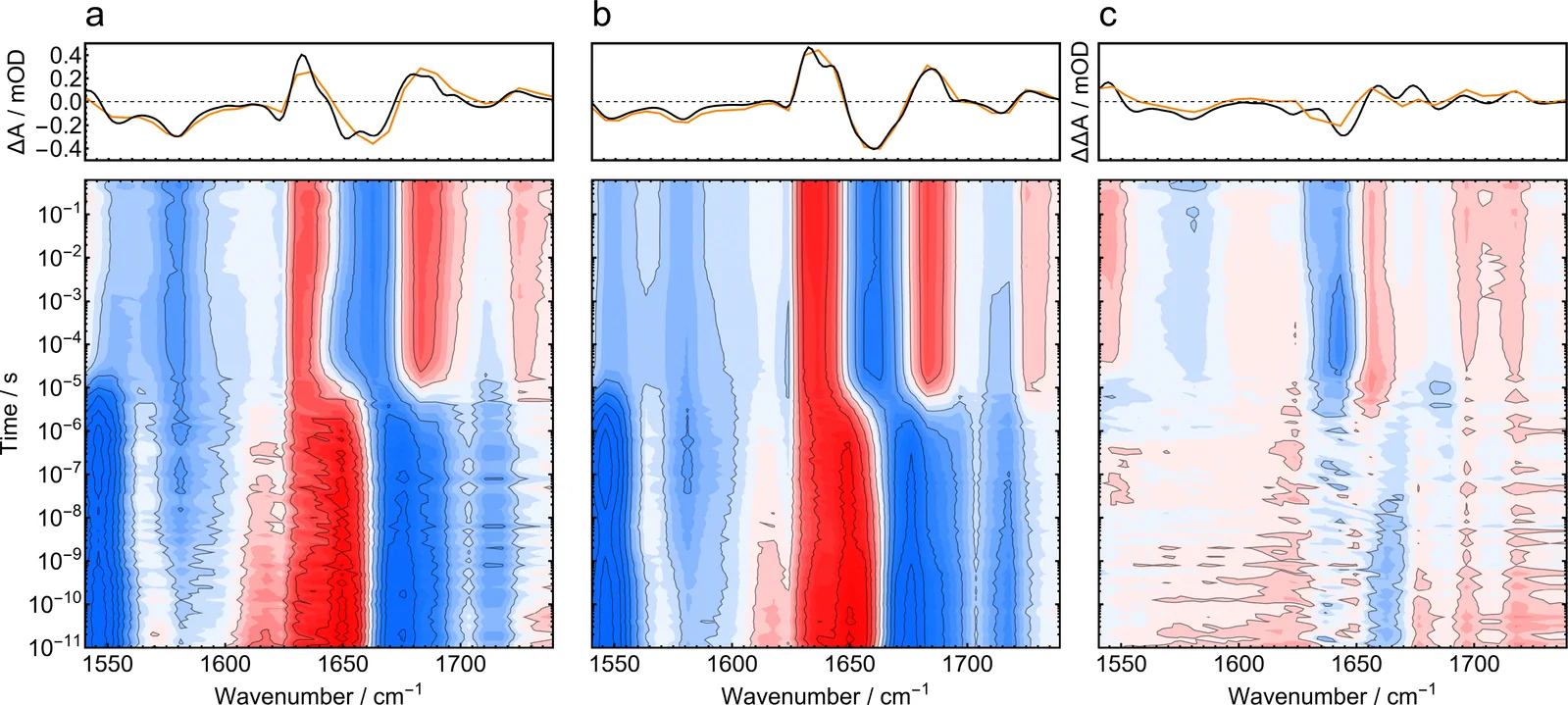
Transient IR data of (a) EL346, (b) LOV_(EL346)_ and (c) the double-difference EL346–LOV_(EL346)_. Colors in the contour plots correspond to positive (red) and negative (blue) transient absorption values. In the plots above, orange lines correspond to the last transient IR spectrum, black lines correspond to the steady-state FTIR difference spectrum. In (c), orange corresponds to the last double-difference spectrum, black corresponds to the double-difference between the two steady state FTIR spectra.

### 3.1. Structure changes in the output domain

In order to pinpoint the structural origin of signal contributions in the adduct state, we also studied a truncated construct that only includes the LOV domain, namely LOV_(EL346)_. The transient IR spectra of LOV_(EL346)_ are shown in Fig. 2b. Global fitting yielded the time constants *τ*_1_ ≈ 4 ns and *τ*_2_ ≈ 6 µs, which is in reasonable agreement with the time constants in the full-length protein. To isolate signal contributions from the output domain of EL346, we normalized each dataset to the bleach at 1546 cm^−1^ to account for differences in the number of molecules excited, and subtracted the LOV dataset from the full-length dataset. We thus obtained a transient double-difference dataset (Fig. 2c), as recently shown for the dimeric LOV-histidine kinase YF1.^16^

The spectra of the singlet and the triplet state cancel out to a high degree, indicating that the photophysics of the RBF cofactor are largely unchanged between the full-length and the LOV-only construct. The small residual signals likely originate from small differences in protein conformation, when the LOV domain is part of a multidomain protein *versus* when it is not. During the adduct formation step at roughly 10 µs, pronounced double-difference signals arise, marking the first structural changes in the effector domain. In other LOV systems, downstream conformational changes are commonly observed at the same time as cysteine adduct formation. The adduct formation is understood as a rate-limiting step, which is followed by a fast structural response of the protein, such that the two molecular events are observed as one.^5,7,14,16,31^

The most prominent features in the double-difference data are a negative signal at ∼1643 cm^−1^ and a positive signal at ∼1656 cm^−1^, which falls into the spectral region of characteristic frequencies of α-helices,^32^ suggesting that a mostly α-helical rearrangement takes place in the output domain during the adduct formation step. This rearrangement is most likely localized in the DHpL domain, which is fully α-helical, and possibly also involves the α-helical interface between the DHpL and CA domains. This assignment is in good agreement with the proposed mechanism in the literature, where interactions in the LOV-DHpL interface and the DHpL-CA interface are modulated by light-activation.^22,24^ Further, smaller positive signals are found at 1546 cm^−1^, 1697 cm^−1^ and 1718 cm^−1^, together with a negative signal at roughly 1580 cm^−1^, which are confirmed by the double-difference of the steady-state FTIR spectra in Fig. 2c. These signals point to additional structural elements that undergo conformational changes during adduct formation, or to different conformational constraints on the LOV domain between the full-length EL346 and the truncated LOV_(EL346)_. While an unambiguous assignment to β-sheets, turns and disordered secondary structure elements is not possible, the high frequencies of the 1697 cm^−1^ and the 1718 cm^−1^ signal could indicate the involvement of carboxyl groups of aspartic and glutamic acid sidechains,^32–34^ or possibly even the amide groups of asparagine and glutamine sidechains,^32,35^ which are abundant in EL346 and could be sensitive to structural changes.

### 3.2. Millisecond process

The EL346 full-length data exhibit another spectral evolution, which appears on a millisecond timescale. It is discernible in the contour plot in Fig. 2a, where the ∼1630 cm^−1^ transient appears to broaden towards higher frequencies, affecting the ∼1660 cm^−1^ bleach. The kinetic is yet more clearly visible in the double-difference data in Fig. 2c. Subtracting the EL346 spectrum before (100 µs) from the spectrum after this process (20 ms) reveals the isolated spectral changes ΔΔ*A*_(20ms–100µs)_ of this millisecond evolution, most prominently featuring a positive signal at 1643 cm^−1^, flanked by two negative signals at 1630 cm^−1^ and 1670 cm^−1^ (see Fig. 3a). This broad spectral signature covers typical frequencies of α-helices, β-sheets, as well as turns and disordered regions, indicating a process that could involve the entire effector domain. Further, small positive signals are present at 1546 cm^−1^, around 1693 cm^−1^ and 1711 cm^−1^, together with a faint negative signal at around 1580 cm^−1^. The high frequencies around 1700 cm^−1^ could correspond to carboxylic acid or amide sidechains. In comparison, calculating ΔΔ*A*_(20ms–100µs)_ for the LOV_(EL346)_ data only reveals a small and featureless baseline offset, indicating that the millisecond process only takes place in full-length EL346, where it is localized in the output domain. To include this process in the global sequential fit of the EL346 data, an additional kinetic step was added, yielding *τ*_3_ ≈ 3 ms. The evolution-associated difference spectra (EADS) resemble the 100 µs and 20 ms spectra (Fig. S1), and reproduce the spectral change between the last two EADS during the millisecond process (Fig. S2). In order to determine whether subsequent kinetic processes take place after 600 ms, we carried out a rapidscan measurement covering 37 ms to 10 s (Fig. S3), where no further spectral evolution was detected. Therefore, we conclude that the activation of EL346 is likely completed with the 3 ms process.

**Fig. 3:**
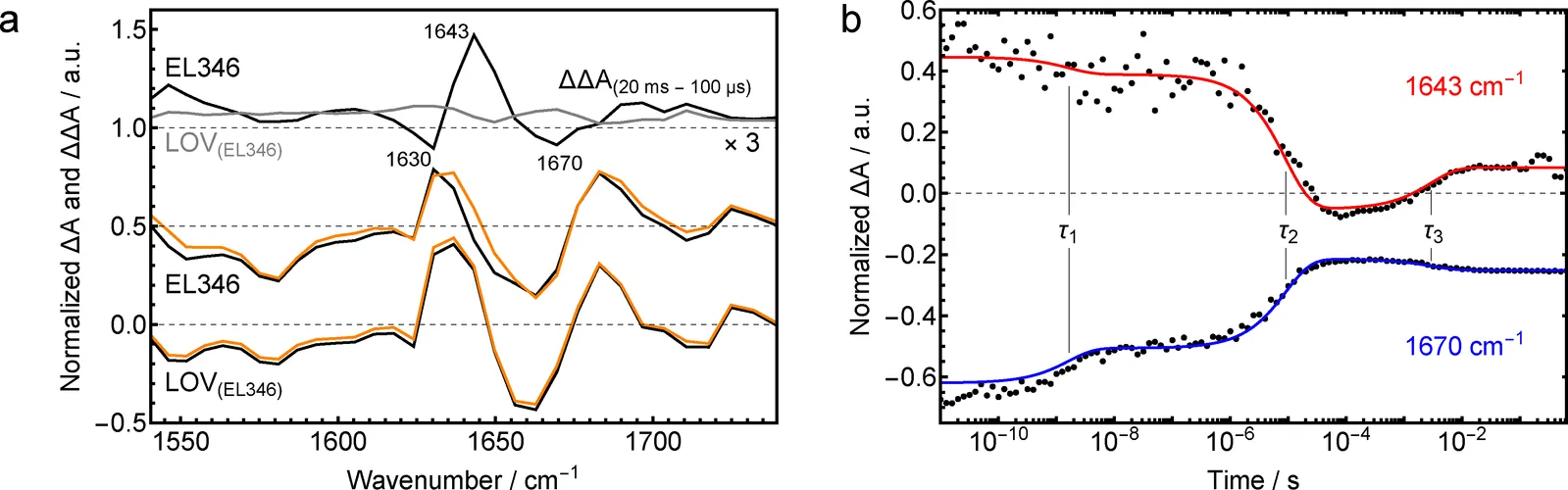
(a) Transient IR spectra at 100 µs (black) and 20 ms (orange) of EL346 and LOV_(EL346)_. The double-difference spectra ΔΔ*A*_(20ms–100µs)_ for EL346 (black) and LOV_(EL346)_ (gray) were multiplied by a factor of 3 for comparison. (b) Exemplary transient absorption traces of EL346 at 1643 cm^−1^ and 1670 cm^−1^. The measured transient IR data are shown as black dots, the global fits of the traces is shown as blue and red lines, respectively.

### 3.3. Effect of AMP-PNP

ATP and its analogue AMP-PNP were shown to stabilize binding of the CA domain to the DHpL domain by contributing a hydrogen-bonding interaction through the γ-phosphate of the nucleotide.^22,24^ Thereby, they tilt the equilibrium between active and inactive conformations, both in the dark and in the light state.^24^ To investigate whether the stabilizing effect of AMP-PNP has consequences for the activation kinetics and mechanism, we measured transient IR spectra of EL346 with added AMP-PNP and MgCl_2_ (Fig. 4). We identified the same processes as without AMP-PNP, with a global fit yielding largely unchanged time constants of *τ*_1_ = 1.5 ns for ISC, *τ*_2_ = 9.5 µs for adduct formation, and *τ*_3_ = 2.6 ms for the millisecond process. The ΔΔ*A*_(20ms–100µs)_ double-difference spectrum of this process gave a signature similar to that without AMP-PNP in Fig. 3a within signal-to-noise, showing a clear peak at 1643 cm^−1^, only the negative signals on either side of it are not as clearly pronounced. These spectral changes are reproduced by the EADS from the global fit (Fig. S7 and S8). Close comparison of the transient spectra at 100 µs and 20 ms in Fig. 3a and 4a might indicate that the conformational changes are slightly altered in the presence of AMP-PNP. We would assess these differences to be minor at most, suggesting that the activation pathway and timescales remain unaffected by AMP-PNP within detectability.

**Fig. 4:**
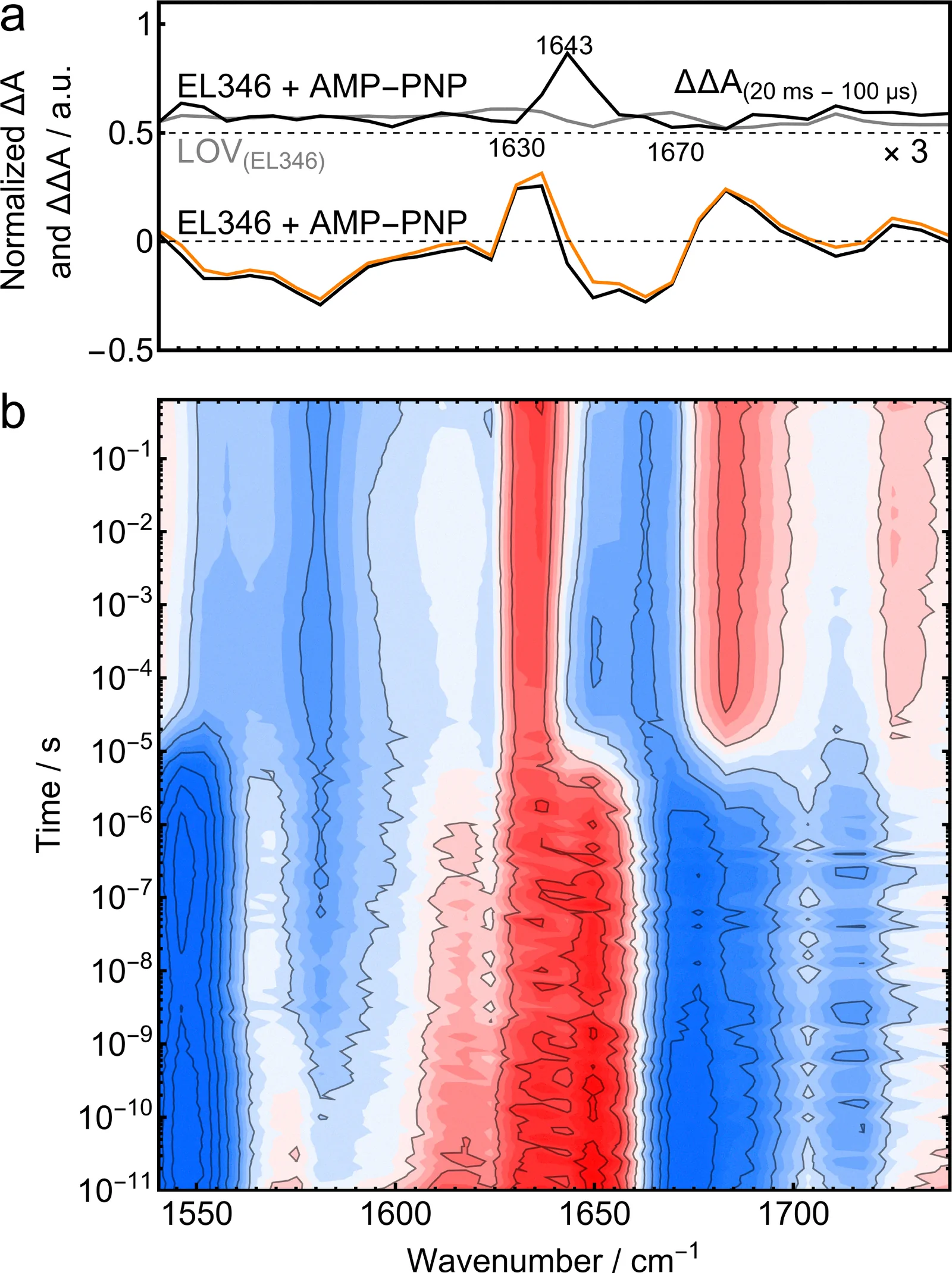
Transient IR data of EL346 with AMP-PNP. (a) Spectra at 100 µs (black) and 20 ms (orange). The difference ΔΔ*A*_(20ms–100µs)_ between the two is shown in black, together with the difference ΔΔ*A*_(20ms–100µs)_ for the LOV_(EL346)_ data in gray. The ΔΔ*A*_(20ms–100µs)_ spectra were multiplied with a factor of 3 for better comparison in this plot. (b) Contour plot of the transient IR data of EL346 with AMP-PNP.

## 4. Discussion

LOV domains are found as part of different multidomain architectures, where they enable signaling through different mechanisms.^1^ It is instructive to compare the spectroscopic response of EL346 to that of YF1 and *As*LOV2, which represent two well-studied reference points of LOV signaling. EL346 bridges the two architectures by sharing a common histidine kinase domain with the dimeric YF1, while exhibiting high structural similarity to the α-helical transducer of *As*LOV2, which is bound directly to the β-sheet surface of the LOV domain.

### 4.1. Histidine kinase signaling in EL346 and YF1

The two LOV-histidine kinase proteins EL346 and YF1 (shown in Fig. 1b and c) both mediate signaling from a LOV domain (gray) through an α-helical transducer (blue) to a CA domain (orange). Notably, YF1 is dimeric and its Jα-helices extend away from the LOV domain to form a coiled-coil linker, whereas EL346 is monomeric, with its α1 and α2-helices bound on the β-sheet surface of the LOV domain.

We compared the obtained EL346–LOV_(EL346)_ double-difference spectra to the double-difference spectra of YF1–*Bs*LOV from our previous work^16^ (see Fig. 5b, c, e and f). Strikingly, both double-difference datasets feature a bleach/transient pair that arises concomitantly with adduct formation. It is located at 1632(−)/1643(+) cm^−1^ in YF1 and ∼1643(−)/1656(+) cm^−1^ in EL346, where the signal later undergoes an additional evolution.

**Fig. 5:**
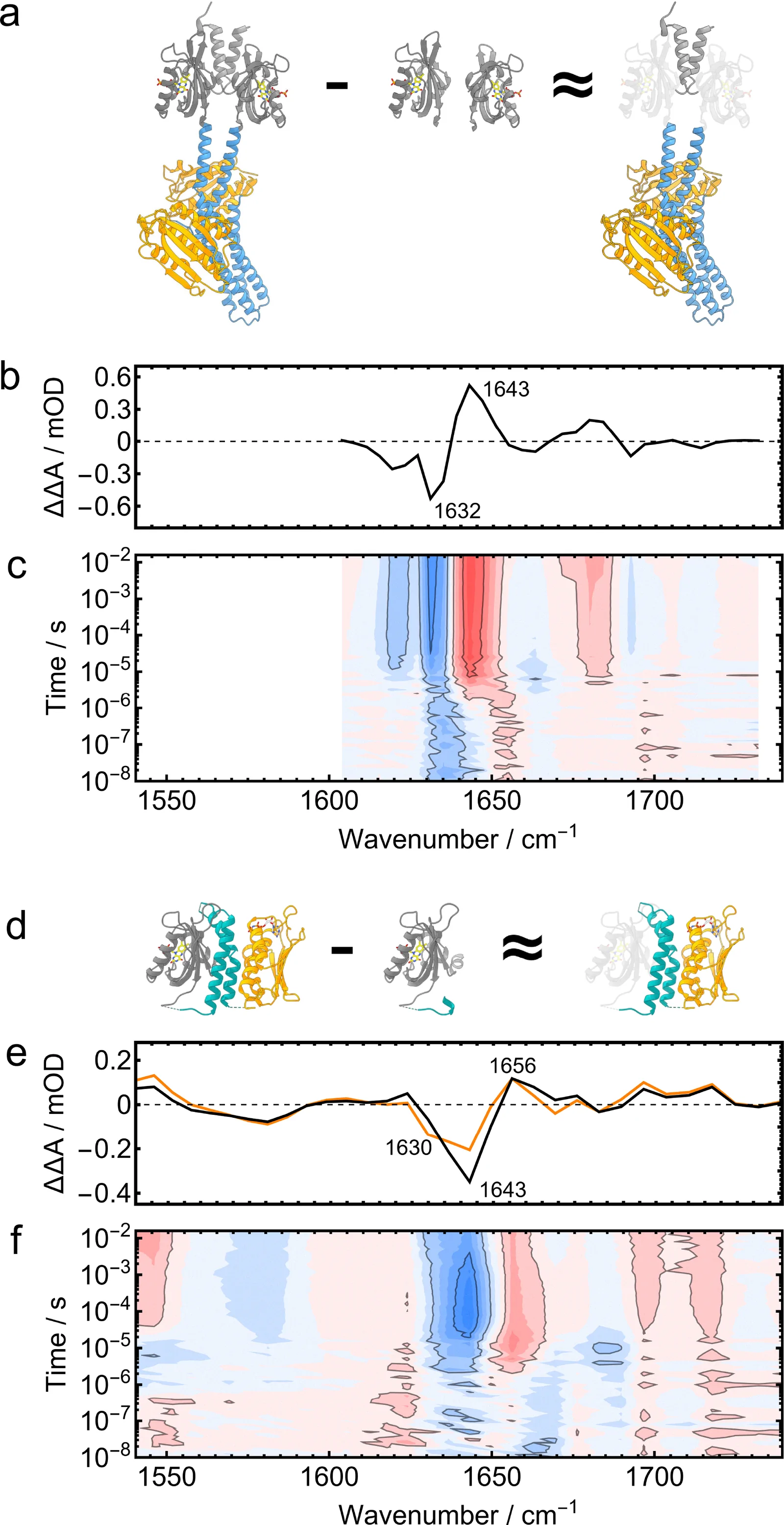
Double-difference data were calculated by subtracting transient IR data of an isolated LOV domain from data of the corresponding full-length LOV multidomain protein. The remaining signal contributions after subtraction are visualized on the crystal structures of (a) YF1 (PDB: 4GCZ) and (d) EL346 (PDB: 4R3A), with signals from the LOV domains mostly subtracted out, and signals from the effector domains prevailing. The double-difference data of YF1 (YF1–*Bs*LOV) is shown in (c), together with the spectrum at 100 µs (b). The openly available data of YF1 were taken from ref. 16 and plotted again for comparison. The double-difference data of EL346 (EL346–LOV_(EL346)_) is shown in (f), together with the spectra at 100 µs (black) and 20 ms (orange) in (e).

In YF1, the frequency of the 1632(−) bleach matches typical observed frequencies of solvent-exposed helices,^6,16^ while the negative/positive signature caused by a blueshift of α-helical species points to supercoiling of α-helices, decreasing the solvent exposure of backbone CO groups.^16,36^ In comparison, the higher frequency of the ∼1643(−) cm^−1^ bleach in EL346 can be interpreted in terms of the DHpL domain being less solvent exposed in the dark state than the Jα-helices in YF1, due to being occluded by the LOV domain and the CA domain on either side. Further, transition dipole coupling to the neighboring structure motifs likely affects the frequency of the DHpL domain.^32,36^ However, the blueshift of α-helical species that underlies the ∼1643(−)/1656(+) cm^−1^ difference signature does not offer a clear interpretation, as the activation of EL346 has been experimentally shown to increase the solvent exposure of the DHpL helices,^22^ which would cause a redshift due to increased H-bonding.^32^

We speculate that the observed shift could be caused by an interplay between two opposite effects. If the DHpL domain partially loses its defined α-helical structure and dissociates from the LOV domain, loss of intra- and inter-helical dipole coupling and loss of H-bonding would, alone, appear as a blueshift. This could then be counteracted to some degree by a gain of solvent accessibility and H-bonding to water molecules, providing a redshift contribution.^32,36^ Due to the competition between these different effects, the precise origin of this α-helical signature remains elusive, and complementary experiments such as small-angle X-ray scattering are required to obtain a more detailed picture of the underlying structural behavior of the DHpL and CA domains.

### 4.2. *As*LOV2-like signaling

The domain architecture of EL346 is somewhat analogous to *As*LOV2, a LOV-domain protein that constitutes a part of phototropin.^22,37^ In *As*LOV2, the LOV core domain binds to the C-terminal Jα-helix and the N-terminal A′α-helix at its β-sheet interface. In EL346, the equivalent binding site on the β-sheet surface is occupied by the α1-helix and the N-terminal A′α-helix, but also binds the α2-helix. On the basis of this structural similarity it was previously speculated that the activation mechanism of EL346 could be similar to that of *As*LOV2.^24^ In *As*LOV2, light activation of the LOV domain leads to downstream conformational changes in the entire protein that culminate in the complete unfolding of the Jα-helix.^4,7^ Mechanistically, the FMN adduct formation in *As*LOV2 causes the sidechain of a nearby glutamine (Q513) to flip, which thereby acts as a lever to perturb the β-sheet surface of the LOV domain and destabilize the Jα and A′α-helices, finally causing them to fully unfold (see Fig. 6).

**Fig. 6:**
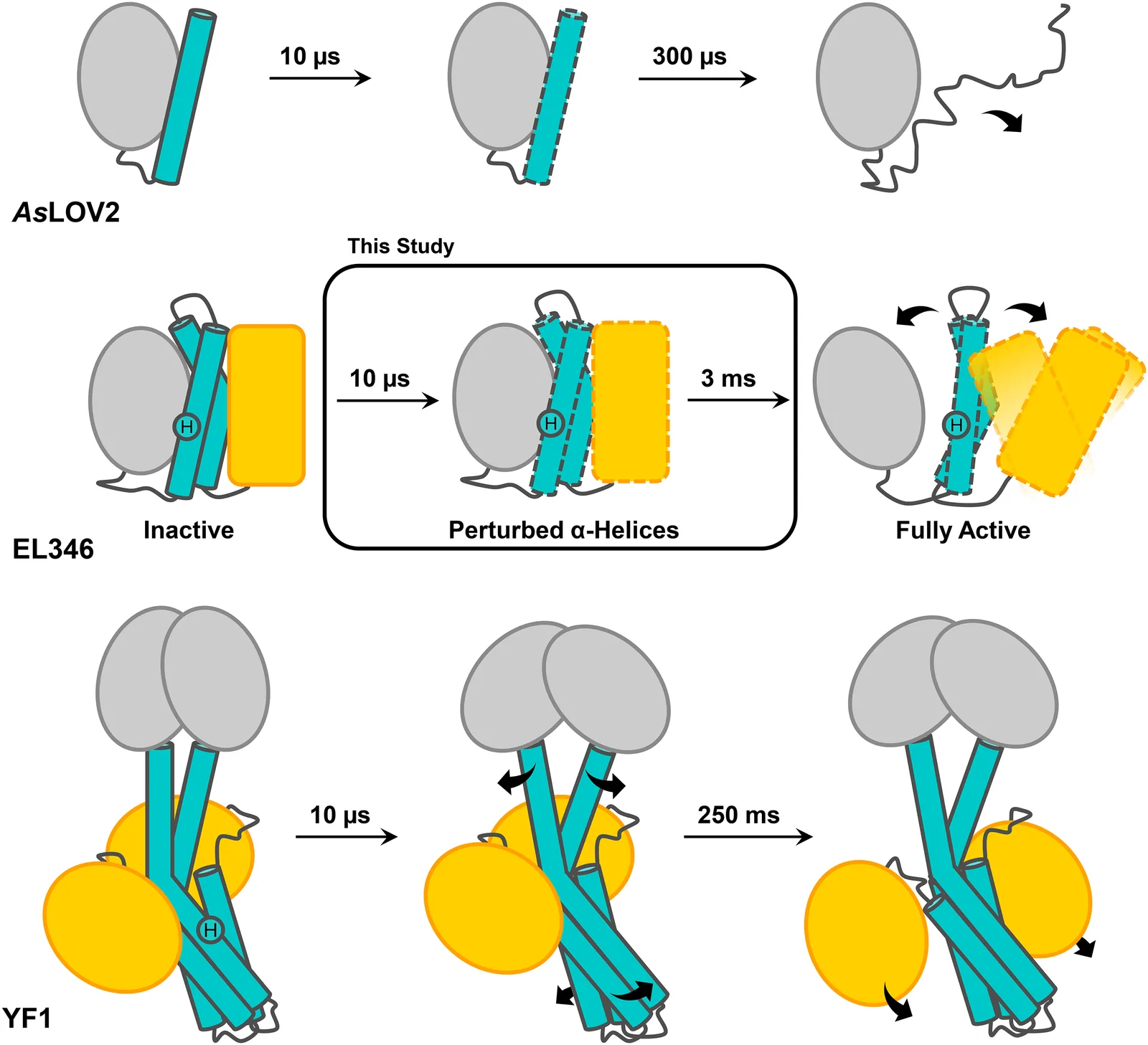
Comparison of the known activation mechanism of *As*LOV2 and YF1 with our proposed activation mechanism of EL346, shown as cartoon structures. The proposed structure changes of EL346 are currently not known with atomic details. Our proposed mechanism features structure changes in the LOV domain during adduct formation at 10 µs, together with α-helical perturbations in the DHpL domain and possibly the CA domain. During the millisecond process, another structural change adapts EL346 to its fully activated state.

The Q513 lever flip in *As*LOV2 is observed together with adduct formation and already couples to structural changes in the Jα-helix at ∼10 µs,^5,6^ matching our observation of α-helical changes concurrent with adduct formation, as extracted from the double-difference spectra (see Fig. 2c). Subsequently, the full Jα-unfolding in *As*LOV2 takes place on a ∼300 µs timescale, exhibiting a pronounced IR signal, where the spectrum experiences a spectrally broad decay that affects signals at 1626 cm^−1^ and 1636 cm^−1^, together with a rise appearing in the region around a 1663 cm^−1^ peak.^5,6^ In the EL346 data, a spectral rise during the millisecond process is present at 1643 cm^−1^, yet the large, pronounced decay observed in *As*LOV2 at lower wavenumbers has no comparable counterpart in the EL346 data. Overall, the changes we observe for EL346 on a millisecond timescale are comparatively smaller than those in *As*LOV2, amounting to ∼30% of the adduct state difference signal (see Fig. 3a). This suggests a less extreme conformational change, thereby questioning whether complete unfolding of DHpL domain helices could be taking place in EL346. In *As*LOV2, mutations at the β-sheet-Jα-interface, as well as truncations and extensions in both terminal helices of the LOV domain have been shown to modulate the timescale and magnitude of the Jα-unfolding signal, allowing production of a wide range of signaling outputs.^5,37^ Therefore, the slow kinetic (3 ms) and small signal amplitude in EL346 place well within the range of possible signaling outputs of *As*LOV2-like signaling, being defined by the unique molecular details at the inter-domain binding interfaces. Further, complete unfolding of the Jα-helix has been proposed to be a truncation artifact.^37^ While the commonly studied *As*LOV2 construct only features the C-terminal Jα-helix, EL346 is a full-length protein that includes the CA domain bound on the DHpL domain, which we speculate likely has a significant effect on the helical dynamics and possibly prohibits complete unfolding of the α1 and α2-helices. Studying truncated constructs that only include the α1-helix, or both the α1 and α2-helices could provide insight into whether CA truly inhibits unfolding of the DHpL domain. The small signal amplitude in EL346 could potentially also be explained by only a small fraction of proteins transitioning to the active conformation. Since the DHpL and CA domains of EL346 have been shown to exist in an equilibrium between enzymatically active and inactive conformations,^24^ the small spectral change during the millisecond process could, in part, be explained by the fact that the equilibrium is tilted only slightly. The millisecond process is the last kinetic observed in our data, and therefore must adapt EL346 to its fully activated state, in which the DHpL and CA domains are fully dissociated, and high domain mobility enables enzymatic activity of the HK output domain.^22,24^ However, in the present data the dissociation event cannot be assigned definitively, as a redshift of α-helical species caused by the expected solvation gain remains spectrally hidden among other effects. On the basis of *As*LOV2,^7^ where the Jα-helix is perturbed during adduct formation and unfolds in a separate kinetic, we hypothesize that the millisecond kinetic could correspond to this final dissociation step of the DHpL and CA domains in EL346 (Fig. 6).

## 5. Conclusion

Here, we presented transient IR data of the full-length LOV-histidine kinase protein EL346. We revealed its activation mechanism following photoexcitation, intersystem crossing, and adduct formation. Through measuring transient IR data of the isolated LOV domain of EL346, we generated double-difference spectra, revealing that α-helical structures of the DHpL and possibly the CA domain are perturbed at the same time as adduct formation at 10 µs. Together with previous double-difference spectra of YF1, we demonstrate that this approach can be highly useful for revealing the distinct spectral signatures indicative of the different activation mechanisms of LOV proteins and potentially other photosensor proteins. In EL346, a subsequent 3 ms process modulates the α-helical signals further, most likely fully adapting the DHpL and CA domains to the final conformational state. Overall, our finding of two protein structure transitions generally matches other LOV signaling modes. Adduct formation is typically the first step where the excited chromophore couples to protein conformational changes, which is in multidomain proteins often followed by further, large scale conformational changes that adapt the protein to its fully active state.^9,14,16,31^ The precise structural details of the transitions during the adduct formation and the millisecond process in EL346 remain unresolved, and it is not yet clear when the domain dissociation event takes place. Together with the common structural theme of a LOV-helix interface, our results place the activation mechanism of EL346 closer to *As*LOV2-like signaling than to the coiled-coil rotation mechanism of the dimeric LOV-histidine kinase YF1, while demonstrating that the extent and kinetics of the responses of α-helical transducers are highly variable and strongly architecture-dependent.

## Author contributions

Philipp Janke: conceptualization, methodology, investigation, validation, formal analysis, resources, data curation, visualization, project administration, writing – original draft. Raoul E. Herzog: methodology, investigation, validation, writing – review & editing. Philipp J. Heckmeier: writing – review & editing. Peter Hamm: conceptualization, methodology, validation, resources, data curation, supervision, project administration, funding acquisition, writing – review & editing.

## Conflicts of interest

There are no conflicts to declare.

## Supplementary Material

CP-028-D6CP03453E-s001

## Data Availability

The data shown in this article are openly available on Zenodo (https://zenodo.org/records/23499332). Supplementary information (SI) is available. See DOI: https://doi.org/10.1039/d6cp03453e.
