# Supplementary material for: Sequential structural changes in the monomeric LOV histidine kinase EL346 revealed by transient infrared spectroscopy
Source: Phys Chem Chem Phys. 2026 Oct 22. Online ahead of print. doi: 10.1039/d6cp03453e (PMC13690264; doi:10.1039/d6cp03453e)
Supplement: Supplementary file 1 [file CP-OLF-D6CP03453E-s001.pdf]

Supplementary Information (SI) for Physical Chemistry Chemical Physics.
This journal is © the Owner Societies

Supporting Information

Sequential Structural Changes in the Monomeric LOV Histidine Kinase EL346 Revealed by Transient Infrared Spectroscopy

Philipp Janke,*[a] Raoul E. Herzog,[a] Philipp J. Heckmeier,[a] and Peter Hamm*[a]

[a] *Department of Chemistry, University of Zurich, Winterthurerstrasse 190, CH-8057 Zurich, Switzerland*
Corresponding authors: philipp.janke@chem.uzh.ch, peter.hamm@chem.uzh.ch.

1 Global Fitting and Evolution-Associated Difference Spectra (EADS)

Table S1 Fitted time constants for the transient IR data using a global fit to a sequential model. ISC quantum yields were estimated from the disappearance of the bleaches at the 1670 cm^{-1} and 1677 cm^{-1} pixels, according to previously described procedures.[1–3] The resulting values are comparable to typical values for other LOV domains.[3]

	τ_1 / ns	τ_2 / μs	τ_3 / ms	Φ_{ISC} / %
EL346	1.6	9.1	2.9	(85 ± 10)
$LOV_{(EL346)}$	3.9	5.9	-	(80 ± 10)
EL346+AMP-PNP	1.5	9.5	2.6	(85 ± 10)

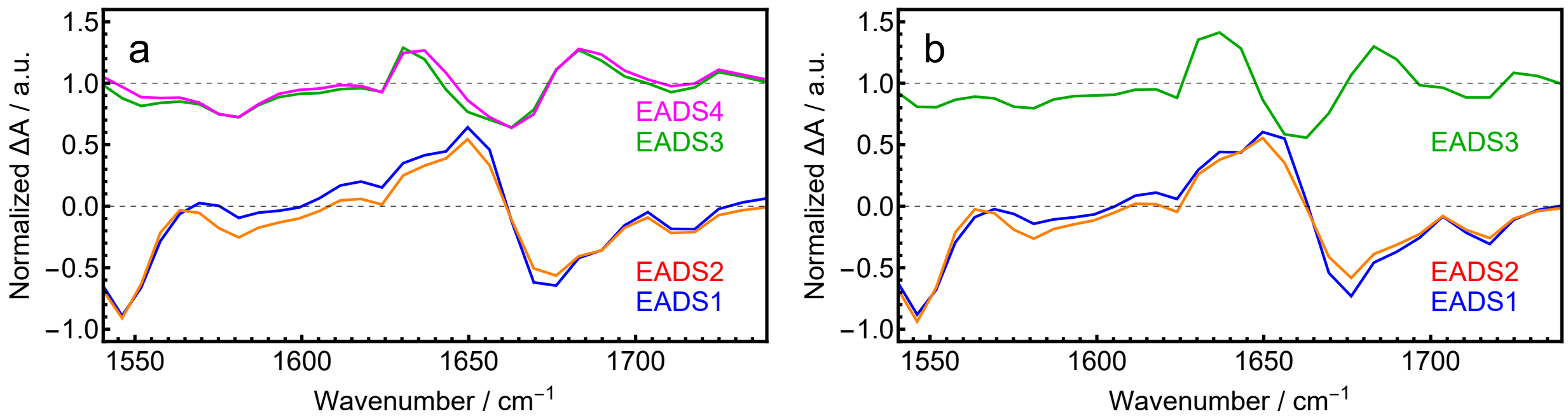

Fig. S1 All EADS from global fitting to a sequential model, for the transient IR data of (a) EL346 and (b) $LOV_{(EL346)}$.

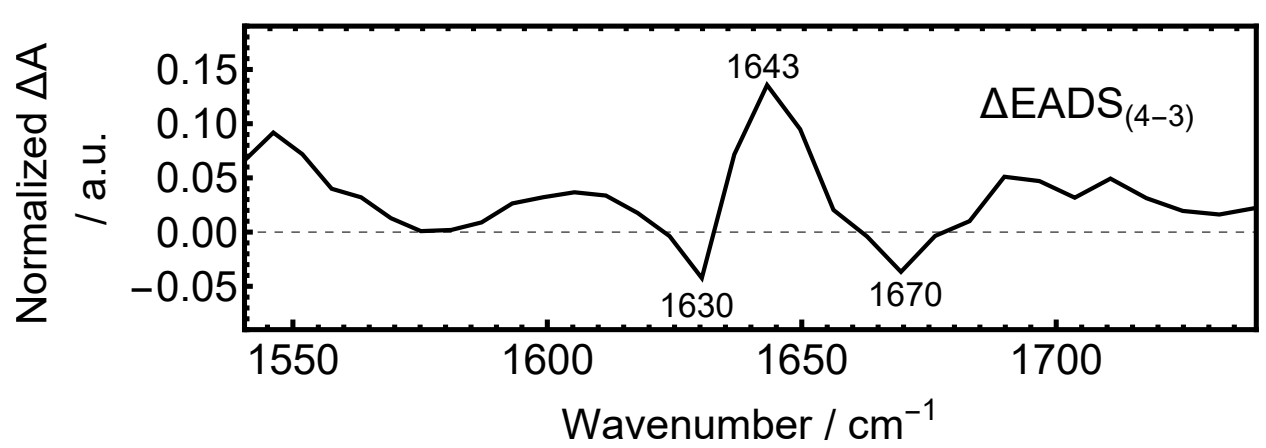

Fig. S2 Taking the difference between the last two EADS of EL346 in Figure S1a produces the double-difference signature $\Delta EADS_{(4-3)}$ of the millisecond process, which closely reproduces the $\Delta\Delta A_{(20\ ms-100\ \mu s)}$ in Figure 3a in the main paper.

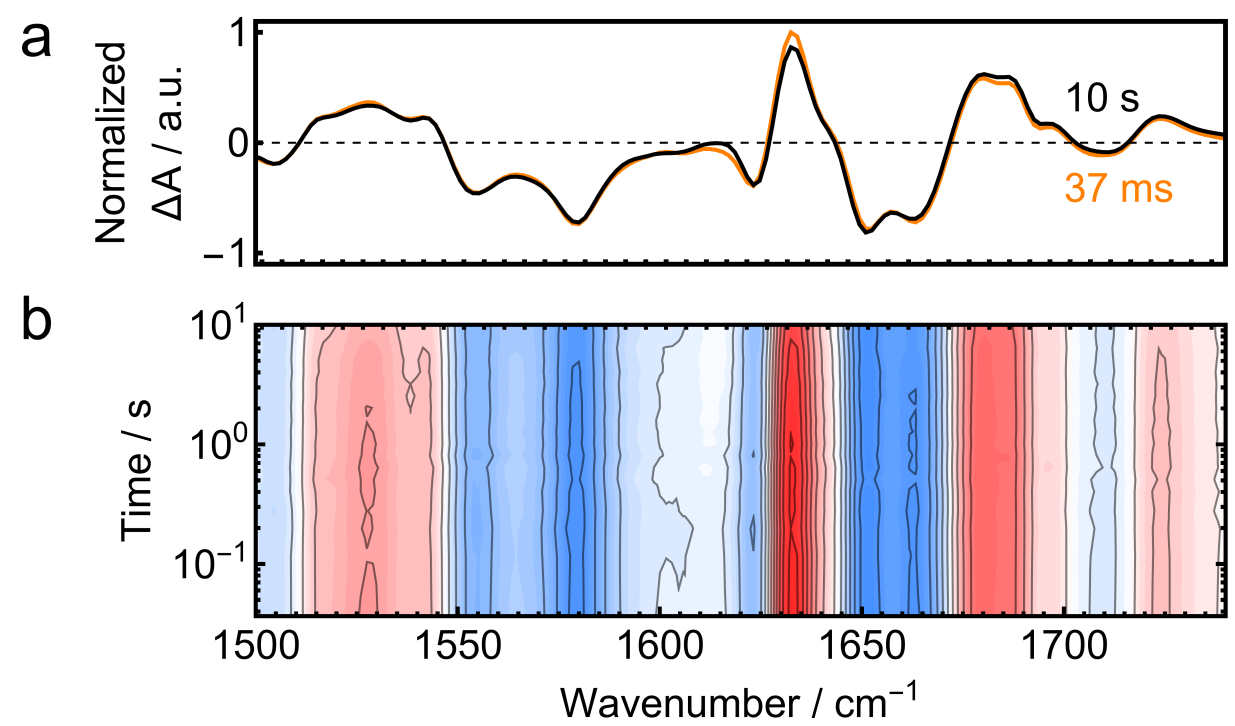

Fig. S3 Rapidscan FTIR spectra of EL346 following blue-light excitation, covering 37 ms to 10 s. Since the data are measured on a single instrument, they are self-consistent and can serve as a control whether a late kinetic process takes place on this timescale. (a) The 37 s spectrum (orange) and the 10 s spectrum (black) are shown. To correct for excited sample flowing out of the IR spot as well as baseline fluctuations, the 10 s spectrum was fitted to the 37 ms spectrum by multiplication with a scaling factor a and addition of a constant offset b, giving $a = 1.09$ and $b = 0.03$. The spectra agree closely with each other, indicating that no further structural adaptation takes place on this timescale. (b) A contourplot of the raw data is shown. Rapidscan data were measured with the same instrumentation as the steady-state FTIR spectra. Data were binned and averaged into 10 bins per decade, which are logarithmically spaced in time.

2 Circular Dichroism Spectroscopy on EL346

Circular dichroism (CD) spectra were measured on a Chirascan V100 (Applied Photophysics). Samples were diluted to approximately 0.3 μM and measured in a 1 cm cuvette at room temperature, with 20 repetitions of 280 nm to 200 nm scans in 1 nm steps, and 0.25 s at each wavelength. Samples were photoswitched by illumination with a 30 mW continuous wave laser at 450 nm (OXLasers, China) for 30 s. Temperature control was realized using a S&A CW3000 T-Series Industrial Chiller, together with a TC 1 Temperature Controller (Quantum Northwest). The CD temperature series was recorded with 1 °C steps and 90 s equilibration time per temperature point.

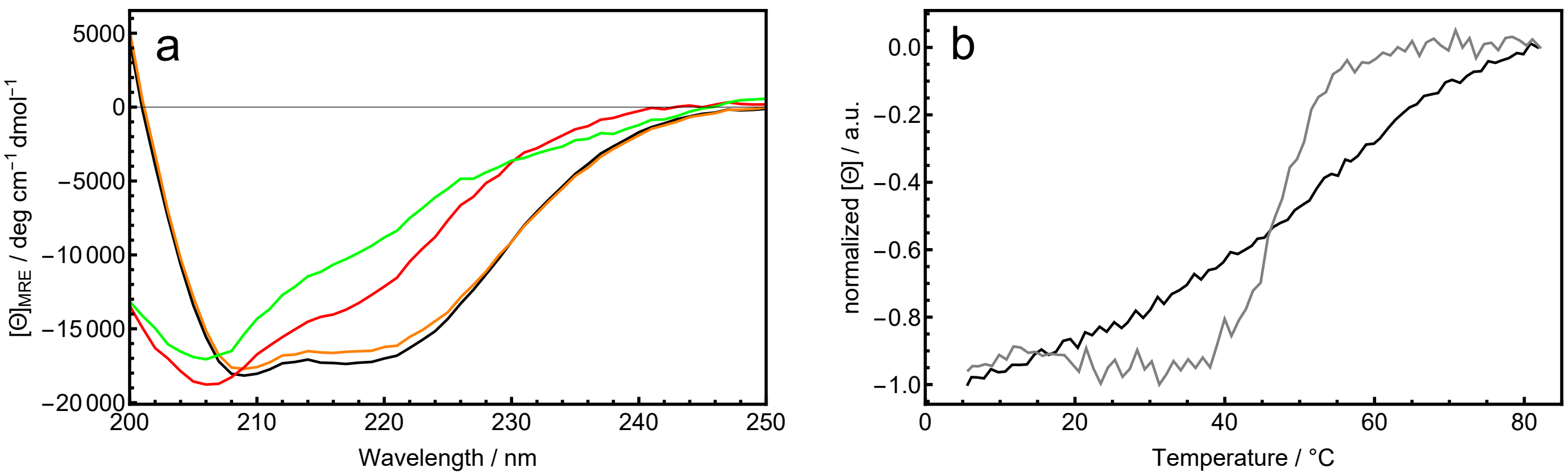

Fig. S4 (a) CD spectra of EL346 in the dark (black) and in the light (orange), $LOV_{(EL346)}$ in the dark (red) and in the light (green). The spectra of the full-length construct are highly similar to previously reported CD spectra for the LOV-histidine kinase YF1,[4] showing a slight decrease of signal between 208 and 222 nm of ca. 5 % upon light activation. The CD spectra of $LOV_{(EL346)}$ show a larger decrease of ca. 10 % in the same region. From this, we conclude that the secondary structure changes in the LOV domain are likely larger than changes in the DHpL and CA domains of the full-length construct. (b) Temperature-dependent CD signal of EL346 (black) and $LOV_{(EL346)}$ (gray) at 222 nm, normalized. $LOV_{(EL346)}$ produced a classical sigmoidal melting curve, whereas EL346 exhibited significantly sloped baselines before and after the main melting point. This could suggest that different inter-domain interactions and different stabilities of the individual domains result in a more complex melting sequence, possibly with contributions from aggregation of partially melted protein.[5,6] Further, a sloped baseline at temperatures lower than the main melting point was previously observed in literature for the LOV protein *Rs*LOV,[7] where it was interpreted as successive loss of α-helical content, suggesting partial unfolding of the helical dimerization interface of *Rs*LOV.[7] We hypothesize that a similar effect could be taking place for EL346, partially unfolding the DHpL domain. However, considering the high degree of covolution of different melting contributions in the EL346 curve, we consider this interpretation highly speculative.

3 Characterization of Heating in the transient IR Experiments

Since EL346 showed a temperature dependence in CD spectroscopy, we assessed heating effects in transient IR experiments in order to ensure reliable experimental conditions. We considered both heating from the infrared probe laser as well as from the visible pump laser.

3.1 Heat Deposition from the IR Laser in the transient IR Experiments

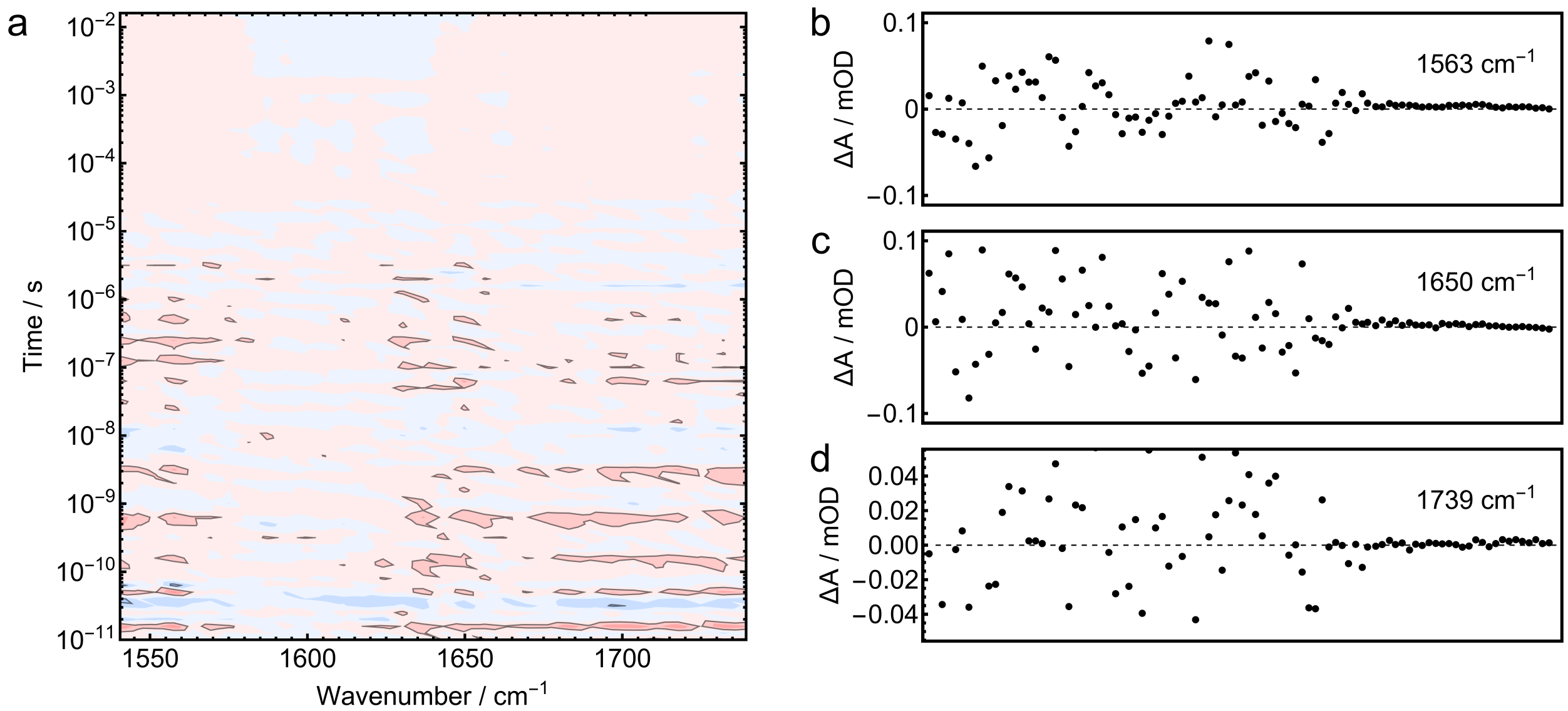

Fig. S5 Transient IR data of EL346, recorded with the visible pump laser blocked. The data are not normalized. A contourplot is shown in (a), with selected traces at different wavenumbers in (b), (c) and (d). The data show an essentially flat baseline at delays later than 10^{-5} s, with higher noise at earlier times. This is a result of these earlier delays being scanned explicitly, while the later delays benefit from binning of individual laser shots, averaging out noise significantly.[8] No baseline artifacts or spectral signatures were observed in this data, indicating that the infrared laser does not cause any thermal contributions or other complications in the transient IR experiments.

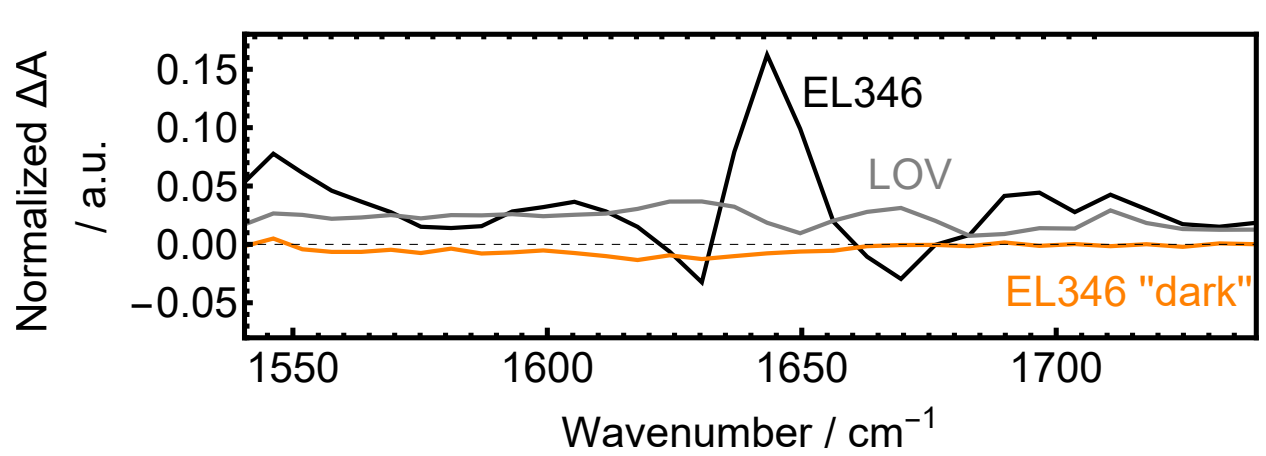

Fig. S6 $\Delta\Delta A_{(20\text{ ms - }100\ \mu\text{s})}$ spectra calculated from the transient IR datasets of EL346 (black), $LOV_{(EL346)}$ (gray) and EL346 "dark" (with a blocked visible pump pulse) (orange). The EL346 and $LOV_{(EL346)}$ are normalized to the largest bleach in the full transient IR dataset, and the EL346 "dark" data is normalized with the same factor as the EL346 data for direct comparability. For EL346, the double-difference signature of the millisecond process is apparent, $LOV_{(EL346)}$ exhibits a small and featureless baseline offset, and EL346 "dark" shows a completely flat baseline, with no indication of a spectral signature or a baseline artifact.

3.2 Heat Deposition from the Visible Pump Pulse in the Transient IR Experiments

If we consider a 1 mM sample that is fully excited by the visible pump pulse in the transient IR experiments, 1 mM ($= 1$ mol/L) photons are absorbed. The energy of a 420 nm photon is $4.73 \cdot 10^{-19}$ J, leading to 285 J/L absorbed energy per volume. Considering the density[9] of D_2O of $\rho = 1.105$ kg/L, this leads to 258 J/kg absorbed energy per mass of solvent. We now assume the extreme case where this absorbed energy is fully converted into heat. The resulting temperature is then calculated using the specific heat capacity[9] of D2O of $c_p = 4200\ \frac{\text{J}}{\text{kg}\cdot\text{K}}$, yielding a temperature change of

$$\Delta T = \frac{258 \text{ J/kg}}{c_p} = 0.061 \text{ K}$$

Realistically, we would need to take into account the fraction of excitation, as well as the adduct formation quantum yields reported for LOV-domains ($\sim$10% to $\sim$50%, with an extreme case at 82%).[3,10] The resulting, more realistic temperature change would then be smaller than 0.061 K. In conclusion, neither the infrared probe laser nor the small temperature change from the visible pump laser contribute significantly to heating in the transient IR experiment. Therefore, we assess the EL346 and $\text{LOV}_{(\text{EL346})}$ transient IR data and the spectral changes therein to be unaffected by temperature changes.

4 Effect of AMP-PNP

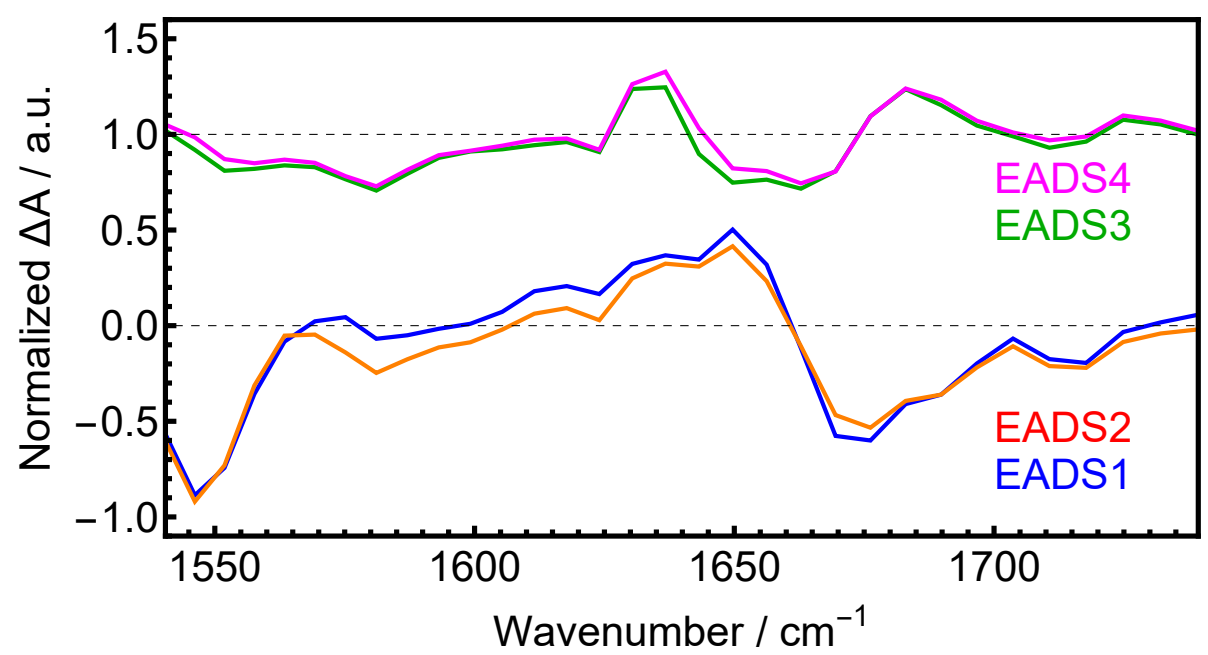

Fig. S7 EADS from global fitting to a sequential mechanism of the transient IR data of EL346 with added AMP-PNP.

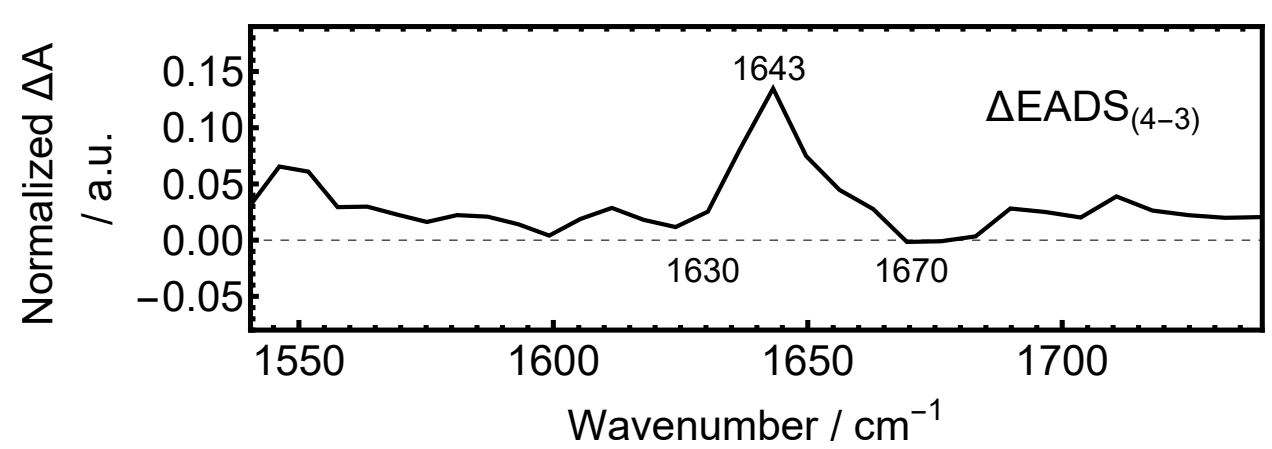

Fig. S8 Taking the difference between the last two EADS of EL346 with AMP-PNP in Figure S7 produces the double-difference signature $\Delta\mathrm{EADS}_{(4-3)}$ of the millisecond process, which closely reproduces the $\Delta\Delta\mathrm{A}_{(20\ \mathrm{ms}-100\ \mu\mathrm{s})}$ in Figure 4a in the main paper.

References

1 M. T. Alexandre, T. Domratcheva, C. Bonetti, L. J. van Wilderen, R. van Grondelle, M.-L. Groot, K. J. Hellingwerf and J. T. Kennis, *Biophysical journal*, 2009, 97, 227–237.
2 R. E. Herzog, P. Janke, P. M. Fischer, P. J. Heckmeier, C. Wei, P. Nag, S. J. Hartmann, M. Mulder, F. Stierli, J. Standfuss *et al.*, *Physical Chemistry Chemical Physics*, 2026.
3 R. E. Herzog, I. F. Harvey-Seutcheu, P. Janke, W. Dai, P. M. Fischer, P. Hamm and P. J. Heckmeier, *Journal of molecular biology*, 2025, 169599.
4 A. Möglich, R. A. Ayers and K. Moffat, *Biophysical Journal*, 2009, 96, 524a.
5 S. M. Kelly, T. J. Jess and N. C. Price, *Biochimica et Biophysica Acta (BBA)-Proteins and Proteomics*, 2005, 1751, 119–139.
6 N. J. Greenfield, *Nature protocols*, 2006, 1, 2527–2535.
7 J. Dietler, R. Schubert, T. G. Krafft, S. Meiler, S. Kainrath, F. Richter, K. Schweimer, M. Weyand, H. Janovjak and A. Möglich, *Journal of Molecular Biology*, 2021, 433, 167107.
8 P. Janke, K. Oppelt, J. Helbing and P. Hamm, *Review of Scientific Instruments*, 2025, 96, 123007.
9 A. H. Harvey, 2012.
10 A. Losi, E. Polverini, B. Quest and W. Gärtner, *Biophysical journal*, 2002, 82, 2627–2634.
